# Recent Works on the Practice of Medicine

**Published:** 1837-10-01

**Authors:** 


### RECENT WORKS ON THE PRACTICE OF MEDICINE.

I. Principles of the Theory and Practice of Medicine; including a Third Edition of the Author's Work upon Diagnosis. By *Marshall Hall*, M.D. F.R.S. L. & E. &c. &c. &c. Octavo, pp. 503. London, 1837.

II. Elements of the Practice of Physic; presenting a View of the present State of Special Pathology and Therapeutics. By *David Craigie*, M.D. F.R.S. E., &c. &c. &c. Octavo, pp. 952.

The objects of the two Authors, whose works we are now to notice, varying in comprehension—Dr. Hall attempting only "to lay down the First Principles of the Theory and Practice of Medicine for the *Medical Student*, and for the *Young Practitioner*," and that too in so condensed a form, as to render the acknowledgment necessary that he "may have omitted some things which had been better inserted"—and Dr. Craigie designing his present work "to embody, in one uniform, harmonious, and comprehensive system, the whole of the theoretical principles, and the practical applications of which the art of preventing and curing diseases now consists," (*preface, p.* vi.)—will at once account for the difference of size in the two compositions. Dr. Hall contrives to condense his "Principles" into a moderate sized volume of only 503 pages, while Dr. Craigie dilates his Elements into a thick and heavy octavo of 952 closely printed pages—with the promise of a second volume, that we may bear in mind that half is not yet told, of all the able author has to tell about the present state of Special Pathology and Therapeutics.

"I wish not to write an unnecessary sentence, or even an unnecessary word," says Dr. Hall, and accordingly, without other note of preparation than the simple announcement, that "the doctrine of inflammation is the most important in the theory of medicine and surgery," *Part* 1, *p.* 3, he enters at once, and somewhat abruptly, upon his subject, with a chapter "on Inflammation," in the very second paragraph of which, however, he pauses to soliloquize upon the "wonderful, he had almost called them sublime, *results* of inflammation," p. 3. But Dr. Craigie, after an explanatory preface of ten pages—ushers in the body of his work by a most elaborate introduction of thirty pages more—in which, after having repeated the definitions of medicine previously received, he adds to them another, viz. his own, which makes medicine "the art of distinguishing, preventing, and curing diseases."—*Introduction, p.* 1.

In like manner, he brings forward the usual definitions of disease only to reject them, and substitutes instead of them, one which makes it "to consist in that change in the properties or structure of any tissue or organ, which renders it unfit for the performance of its actions or functions according to the laws of the healthy frame"—p. 3. "Definitions are dangerous things," and it would not be difficult to shew that a few more such definitions as the above might prove exceedingly dangerous to the reputation for them of even a practised author like Dr. Craigie. The danger to both author and reader, from a vague or inaccurate, or too contracted definition, is, that the one will be always using, and the other understanding terms in another, a more precise, and a more comprehensive sense, than the defined one. And the especial danger of all reformers, in medicine as well as in politics, is that of overlooking the difference between the detection of errors and the development of truth. Not that we would charge Dr. Craigie with the common vice of finding fault, or deny him the rare merit of developing truth. The introduction to his "Elements" is a very sensible and clever exposé of his views of medicine generally, but the value of it is somewhat lessened by the involved and complicated style in which he writes, requiring the closest attention in the reader, and sometimes calling for no little exercise of thought to get at the meaning of the writer. In it, he explains what is to be understood by etiology, nosogeny, nosology, semeiography or nosography, pathology and therapeutics—the divisions of which medicine is considered by him to consist—p. 30. We shall follow him rapidly through each.

1. ETIOLOGY, *or the Doctrine of Causes.* "The formation of diseases is understood to depend on the presence or previous operation of certain circumstances, which are classed together under the general name of causes. The department which undertakes to investigate the nature and operation of these agents, is denominated ETIOLOGY."—P. 13. The usual distinction of causes into remote and proximate is admitted by Dr. C., who asserts, however, that "of these two kinds of causes, definitions in general too abstract and metaphysical have been given; and pathological authors have been too solicitous in their desire to avoid assigning undue influence to either class, to designate them by characters so general, that it is difficult to attach to them any definite idea."—P. 13. In other words, the definitions of those writers who preceded Dr. Craigie, have been so constructed as to define nothing. Of course we have a right to expect something better from himself—and, accordingly, "all those circumstances which are observed to produce in animal bodies sickliness or morbid action, are comprehended under the general name of remote causes."—P. 14. A wide enough definition certainly, as every one must admit. "These (remote causes) are distinguished into predisposing causes, or those which induce a disposition to disease, and exciting or occasional causes, or those which, when the disposition is already established, rouse it to action, and give rise to actual disease."—P. 14. The former include what are denominated *internal* causes, and the diatheses morbosæ of the schools. The latter comprise "all those circumstances and accidents, the operation of which upon the frame is followed more or less directly by the establishment of definite morbid action." P. 18. "Every occasional or external cause may, by habitual, continued, or repeated operation, give rise to effects which constitute morbid predisposition, and hence may become at once an occasional, exciting, or external cause, and a predisposing or internal cause. The best examples of this combination are presented by the effects of terrestrial or miasmatic emanations of marshy, insalubrious, and malarial districts—the effects of residence in tropical and hot climates where the solar heat is intense, and the effects of the habitual use of spirituous, vinous, and fermented liquors."—P. 19. The term " proximate cause of disease," Dr. C. thinks, it would be best to banish entirely from the usage of physicians, and to substitute in its place the term *pathological cause*, or simply *pathological character* of the disease, which would then have a definite signification." Is there then no proximate cause of disease at all, in the true acceptation of the term? Or, is every cause of disease to be resolved into the one general idea of remote causes? If causes are divisable into remote *and* proximate, the change of terms by the banishment of one and the substitution of another, does not render the signification one whit less vague and erroneous. But the Doctor has himself fallen into the very common mistake, which it is exceedingly difficult to avoid, that of applying our own sense to the words of others—and then reasoning against the use of words by their authors, as though their words necessarily meant what we understand by them. Thus, he gets rid of the old idea attached to the term disease, namely, derangement of function; attaches to it the more comprehensive idea of organic lesion as well as functional derangement—and then using the word in his own sense, sets aside another term altogether because of its inapplicability to disease as understood by him, although more applicable to disease in the meaning of the word as used by those who invented the term now to be got rid of—and in his haste to do this, has scarcely time to invent a less objectionable one—for surely " pathological cause" is as objectionablé as " proximate cause"—when applied to disease itself, and not to a cause of disease at all —and although the alternative phrase "pathological character of the disease" may be better in speaking of the disease as existing, it is altogether useless in describing the cause of that disease before it actually exists. We suspect that Dr. Craigie gains little, and are quite sure that the profession will be no greater gainers, by his attempted improvements in medical definition. The students, by whom they are likely to be adopted, will have few clearer notions of disease and its causes—and the practitioners by whom his work may be consulted, will be always in peril of confounding things old and new. We say this, in no spirit of harsh or invidious criticism—for Dr. Craigie's volume is a very valuable one, and can well afford to submit its excrescences to the pruning knife.

2. Nosogeny—or the formation of Diseases, Νοσος Γενος. " The only legitimate mode of forming a correct theory of the formation of diseases, is to study the operation of remote causes generally, to observe their effects on the human body and its organs, and to endeavour to trace the connexion between the operation of these causes and the morbid changes induced in the course of various diseases."—P. 21.

3. Nosology. " With the view of communicating just ideas of the resemblances and affinities, as well as the differences of diseases, and exhibiting correct views of their mutual relations, it is usual to distribute diseases in a certain method or order."—P. 22.

"The general principle of nosological distinction is to arrange diseases into certain similar tribes or assemblages, according to their resemblances and their dissimilitudes, in imitation of the arrangements of natural history, &c. In distributing diseases in this manner, it is necessary to distinguish each Class, Order, Family, Genus, and Species, by a certain number of characters, by which it may be known from those which most closely resemble it, and to designate each, thus distinguished, by an appropriate character and appellation. Nosology, may, therefore, be distinguished into two divisions; *Nosotaxy*, or the distribution and classification of diseases, and *Nosonomy*, or the nomenclature of diseases."—p. 23.

"In the following work I propose to adopt, as the basis of classific distinction, the pathological nature of the different morbid processes, and to subdivide these as nearly as may be, according to the anatomical arrangement of the textures and organs of the animal body. On this principle I shall consider in the following order Fevers, Inflammatory Diseases, Hemorrhagic Diseases, Dropsical Disorders, Disorders affecting the properties of many organs at once, not always in a very intelligible manner, Mental Disorders, and lastly, Organic Changes, not referrible to either of these heads."—p. 24.

This department of medicine originated with Sydenham, whose plan was first carried into effect by Sauvages, and improved upon by Cullen and other nosologists, none of whom are deemed worthy by Dr. Craigie of being mentioned with any thing like so large a meed of praise as Dr. Cullen. "While Sauvages has the undoubted merit of first sketching the plan, and exhibiting the arrangement in a degree of wonderful perfection, considering that it was a first attempt, it is impossible to deny that Cullen has effected more important and real improvements, than all the other nosologists who have attempted it; and scarcely those of Young, Pinel, and Mason Good, can be regarded in any other light than as slight and partial improvements on the method and distinctions of Cullen."—p. 23. An opinion from which we must take the liberty of dissenting—Cullen's nosological arrangement is as immeasurably inferior to Mason Good's, whether in method or completeness—as his descriptions of disease are incomparably preferable to those of that lamented physician. The proof is, that Cullen's Nosology is passing out of the hands of the present generation, and will remain only in their heads among the things that were, but are not—whereas, his descriptions of disease, are still, in most cases, the best that can be given. Mason Good's descriptions of disease will pass away, the erudition of that author serving sadly to adulterate the results of his own observation—but, in as much as the basis of his nosological arrangement is more natural than Cullen's, and less arbitrary, it will remain the text book of our medical classes, when that of Cullen is only to be found on the shelves of the learned.

4. NOSOGRAPHY or SEMEIOGRAPHY, or the Department of Practical Medicine which describes diseases according to their symptoms—these being divided into *essential, pathognomic*, or *diagnostic*;—*general*, and *accessory*, but *not necessary*;—and *secondary* or *supervening*, the *symptomata symptomatum* and *symptomata supervenientia* of authors. "It is of the utmost importance for the physician to observe carefully and distinguish accurately these different classes of symptoms; and as this cannot be accomplished without very just and accurate ideas on pathology, general and special, it becomes impossible in practical application to disjoin the knowledge of morbid processes from those (that) of their characteristic symptoms; and the whole object of the study of symptoms is to connect them with their appropriate morbid or pathological causes, &c. If we could always repose confidence in the accuracy of our observation, and in the constancy of the relations established between these diagnostic signs and the pathological state of the organs, that part of medicine which consists in recognizing and distinguishing diseases would then be a perfect art. From this high degree of perfection it is still remote. Though pathological research has assuredly succeeded in diminishing much the number of uncertainties and obscurities between symptoms and their causes, the physician has still daily occasion to remark the fallaciousness of some of his most usual diagnostic means; and the progress of various diseases affords examples of complication and obscurity, which make him feel forcibly the conjectural nature of his art, until symptoms so unequivocal have taken place, that diagnosis is no longer practically useful."—p. 12.

In our notice of Dr. Hall's work, we shall have shortly to recur to this department of medicine. In the meanwhile we pass on to

5. Pathology, or the science which enables " the Practitioner, in any given case or set of cases of disease," " to form from the attentive consideration of the external signs and symptoms, an idea as accurate as possible, of the nature and extent of the morbid action or actions in the tissues and organs of the living body" is rightly judged to be " a science of great extent, and embracing several subdivisions." These are *Pathological Physiology*, or the assemblage of facts obtained by the study and comparison of the actions and functions observable in the healthy body and their variations in the different morbid states of particular organs, or of the system at large. *Pathological Chemistry*, which, from the knowledge possessed of " the natural conditions and chemical constitution of the different fluids of the animal body;" and " of the changes which take place during disease," enables the physician to determine the nature of those changes. And *Pathological Anatomy*, a third species of information, " derived from the careful inspection of the organs of the bodies of those cut off by disease," " regarding the effects, and occasionally regarding the nature of the morbid action."—pp. 5 and 6.

" Pathology and Symptomatology are so closely and necessarily allied, that neither can properly exist alone, or be cultivated apart. Every fact and principle of pathology derives its value from the explanation which it affords of any symptoms or set of symptoms; and it is totally impossible to establish a rational symptomatology without a collection of such pathological facts and principles." Hence " the great object of pathological inquiry is, to enable the physician to distinguish between essential or pathognomonic, and general, or common, or accessory symptoms; and, above all, to determine from the presence of each, the stage, degree, intensity, and effect of the morbid process, with the view to treatment."—pp. 12-13.

6. Therapeutics, comprehending two great divisions—one, Prophylactics, relating to the means of maintaining health and preventing disease; the other, Therapeutics, properly so called. Each embracing two subdivisions: viz:—

| | |
|---|---|
| The Prophylactic or Preventive Department.. | a. Hygiene.<br>b. Medical Police. |
| The Therapeutic or Curative Department.. | a. General Therapeutics.<br>b. Special Therapeutics. |

HYGIENE "relating to the general rules established by Physiology, Etiology and Pathology for the preservation of health." Medical Police referring to "those measures either of precaution, prevention, or rectification, which are adopted to counteract the operation of deleterious principles or morbific agents generally on the human frame."—p. 24-5.

*General Therapeutics,* including "the consideration of the general principles on which the removal of morbid action is to be effected." Special Therapeutics, "the mode of applying these general principles to the treatment of individual diseases."—p. 25.

"If in all instances, the pathological cause or causes were perfectly known, and if at the same time we were perfectly acquainted with agents which could operate on these causes directly and efficiently, and remove them entirely, the principles of therapeutics would rest on a sure and immutable foundation, the curative indications would be simply to remove the pathological cause or causes of the disease, and the healing art would be reduced to great certainty and precision. This perfection, however, pathology has not yet acquired; and the principal lesson which it has taught, is that what is named *cure, consists not in the positive removal or extinction of a morbid process by direct means, but in the gradual subsidence of the morbid action under a favourable combination of circumstances, and the restoration of the action of health.* The cure of disease by direct means is indeed very rarely practicable; and though pathological causes are known, our means do not operate on them; while in the diseases in which the causes are unknown, curative indications must be derived from symptoms. When healthy properties are impaired, we know no agent by which they can be directly restored; when vital action is perverted or deranged, we possess no means of immediately rectifying it, but must be satisfied with using those means under which it is most likely to rectify itself; and when morbid processes are established, they pursue a certain course, and tend to a particular termination, and all that the physician can do, is to moderate and restrain the violence of the process so much, as to prevent it from injuring important and essential organs."—p. 26.

In our notice of Dr. Hall's work we shall be excused from any very close analysis, by the difficulty, almost insuperable, of condensing what has been already condensed to the utmost of the author's ability—and by his own acknowledged inaccuracy of arrangement, arising as a necessary consequence out of his indifference to the severe scrutiny of the Nosologist, for the sake of being "of use to the young clinical student, or practitioner."—p. 240.

The theory of medicine occupies the first part of the volume, and is taken up with several instructive chapters,—1st. On Inflammation, "the most important doctrine in the Theory of Medicine."—2nd. On Tubercle, "after Inflammation the most important of the subjects comprised in the Theory of

Medicine—or Pathology."—p. 42. 3rd. On Melanoses, a disease, "of little importance to the practitioner," for three reasons—it is rare of occurrence, rarely capable of detection, and when detected suggests no indications for the treatment; and although it has recently greatly engrossed the attention of pathologists, Dr. Hall very wisely devotes to it "precisely," and only "that degree of attention which it appears to deserve."—p. 54. 4th. On Encephalosis and Scirrhus. 5th. On Fever, "scarcely less important than the subject of Inflammation,"—p. 60. 6th. On Irritation, as "a subject of great practical importance, but hitherto almost neglected by the profession," 65, and 7th. On Exhaustion, as "another subject almost overlooked by the profession," p. 68. 8th. On Sinking. 9th. On Blood-letting. 10th. On Mercury, and 11th. On the Tartrate of Antimony.

We shall begin at the beginning, with the all-important subject of Inflammation, the *Causes, Signs, Changes, Symptoms, Nature, Modifications, Influence on the System, Diffusion,* and *Treatment of which,* with the distinctions between it and Congestion, and between it and Irritation, are separately considered by Dr. Hall, and, for the most part, satisfactorily although summarily disposed of.

1. The Causes of Inflammation are divisible, according to Dr. Craigie, into two orders, the *Predisponent,* including "a plethoric habit—actually existing, or those circumstances which, either separately or jointly induce a plethoric habit;" "indolent or inactive habits;" "*infancy, youth,* and *manhood; male sex,* and occasionally sexual differences; the Winter or Spring season, and occasionally a peculiar state of the atmosphere:"—*Craigie,* p. 403.—and, the *exciting,* to which last "may be referred all those circumstances which, either applied to, or operating upon the human body, more or less directly, may give rise to a distinct inflammatory attack. Of this kind are extreme cold or intense heat; the application of cold, either in the form of cold air, cold water, or cold drink to the body previously heated; excessive corporeal exertion, so as to induce much heat and sweating, and thereby a temporary febrile state"—"intense and long continued intellectual exertion, or intense mental emotions often accompanied with loss of sleep; articles of food or drink which derange the action of the stomach and bowels; mechanical injuries, as falls, contusions, wrenches; incised, lacerated, or punctured wounds; fractures, simple and compound; dislocations, simple and compound; scalds, burns, and scorchings; various injuries effected by chemical agents, &c.; in short, any cause or causes which produce on the living tissues a strong, intense, or injurious impression."—*Craigie,* p. 404.

Nothing can be looser than the above; Dr. Hall's division into *The Mechanical,* including incision, laceration, contusion, &c.—The *Chemical,* such as heat, cold, electricity, and the operation of acids, alkalies, &c.—The *Vital,* and the *Constitutional,* is greatly to be preferred.

"The principal examples of *internal* inflammation from the operation of chemical causes, are those which have arisen from the attempt to swallow hot water, or acrid fluids. Some part of these fluids may reach the stomach and produce inflammation in that organ; but the principal result is an affection (œdema) of the rima glottidis, with symptoms *resembling* those of *croup.* I first," says the last author "called the attention of the profession to the accident as it occurs in children from drinking out of the spout of the tea-kettle, or tea-pot"—as uncommon a practice, we trust, as it is an uncleanly one. "A similar result followed, in one sad case, the administration of a liniment by mistake for a draught. The physician should be alive to the possibility of such an event, for the *cause* of the affection *might* be concealed by a guilty nurse."—*Hall*, p. 5.

The vital causes of inflammation, appear to be the innate energies of the animal frame, for the preservation and maintenance of life and health in the natural state of the creature, and for the reparation of injuries, when affected by mechanical violence.

The constitutional causes operate in producing furunculi, paronychiæ, erysipelas, gout, &c. Internal inflammation from exposure to cold and wet;—from Rubeola, &c.; and the effects of the Ergot of Rye; are alike produced through the medium of the nervous and vascular, or general system.

Dr. Craigie suspects that "what has been denominated the *Phlogistic Diathesis* is in many instances the general disturbance occasioned by chronic inflammations of a lung or liver, disease of the heart, or disease of the kidney."—p. 16. According to him, also, we have no very distinct proof of the existence of this diathesis, and, of course, as little satisfactory information respecting its exact nature.

II. The Signs of Inflammation, and the Symptoms in Inflammation, occupy distinct sections in Dr. Hall's arrangement, the former being referred to those localities of external, visible, and tangible inflammation, the latter embracing "the affections of the *general* systems, as distinguished from those of the *part* immediately subjected to the disease. When *external*, inflammation displays the well-known signs of *redness, tumor, augmented temperature and augmented sensibility*, the *rubor, tumor, calor, dolor*, of Celsus and the old authors, when *internal*, it requires to manifest itself by symptoms. The *signs* are local, and confined to the part affected—the *symptoms*, general, and more or less affecting the whole constitution.

In those parts of the body only, which are accessible to sensible examination can three of the four events before enumerated be observed or recognized. "The sensations of the patient will announce the pain of which such parts are the seat; but it is impossible to tell whether they are red and swelled or not; and if their temperature is greater than usual, we have no means of knowing either from the sensations of the patient, or from our own observation, or by means of instruments."—*Craigie*, p. 381.

The *redness* in inflammation depends upon the augmented quantity of blood, of the red particles especially; this, upon the increased size of the minute arteries and veins, and of the true capillaries. It "is seen only in the skin, or in some of those points of the mucous surfaces which are accessible to inspection." *Craigie*, p. 381. It varies in degree, being either bright or dull, and is sometimes shaded with brown and purple, this last approaching to lividity; in permanence, sometimes disappearing upon pressure, at others being unaffected by it; and in configuration, being in one case diffused, in another circumscribed.

"Accompanying the redness, and not easily separable from it, is the lustre of an inflamed part." This "may be either diminished and destroyed, or much increased in intensity. In most cutaneous inflammations, in which the redness is bounded by a distinct margin, the lustre is diminished, and the surface dull. In others, however, especially, which attack the lower extremities, as in the *erythema læve* of Dr. Willan, the lustre is augmented, and the whole inflamed surface is smooth and shining. This state of the skin generally indicates great dryness, and much tension."—*Craigie*, p. 382.

The *sense of heat* is much greater than the actual increase of heat itself—arising from the *greater sensibility* of the part, as this latter is probably caused by the greater flow of blood, and augmented still further under the influence of the systole of the left ventricle.

*Inflammatory pain* increases in consequence of pressure, and may thereby be distinguished from spasmodic pains, and the pains arising from simple distention: " —— if the part to which the pain is referred be remote from examination, or cannot be pressed in consequence of being covered by bony or incompressible walls, any motion which has the same effect will increase the pain. Hence the utility of making the patient cough in detecting the presence of inflammation of the brain or its membranes, or inflammation sometimes of the belly, and of making him take a full inspiration in detecting the existence of inflammation of the lungs."—*Craigie*, p. 384.

Pressure may sometimes be necessary in order to the detection of inflammation, where the augmented sensibility of an inflamed part is characterized by tenderness rather than actual pain. " The pit of the stomach is tender during inflammation of the stomach, during fevers, in small-pox, and some other morbid states, in which this organ is affected with squeamishness, sickness, or vomiting. The skin over an inflamed or gouty joint, or a rheumatic muscle, is tender; in various morbid states of the mouth, throat, &c. food cannot be put into it without producing much uneasiness ; and where an abscess is coming near the integuments, a sense of tenderness is almost invariable."—*Craigie*, p. 385.

The *tumor*, or *swelling*, accompanying inflammation, like the heat, and sensibility, is traceable to the increase, principally, of blood in the part: —" *principally*," says Dr. Hall, " because inflammation does not subsist long without more or less of *effusion*, either of *albumen* or *serum*, in recent inflammation ; of albumino-fibrine or lymph in the less recent, or of both; events of vast importance in this sometimes morbid, sometimes curative process."—*Hall*, p. 7.

The general varieties of *swelling* are, the *diffuse*, occurring in inflammation of the membranous tissues, such as the skin, mucous membrane, fascia, capsular ligaments, and synovial membranes :—the *circumscribed*, of which a common phlegmon in the filamentous or cellular tissue, affords the best example :—and the *marginate*, having a distinct edge or boundary, like that of erysipelas, and seen in the *erythema marginatum* of Willan, and in the state of the skin accompanying traumatic gangrenes. Dr. Craigie introduces three others, general, pointing, and compressible, but the first is diffused swelling more widely diffused. The second belongs to the terminations rather than to the signs of inflammation. And the last shews that inflammation has terminated or is terminating in œdema, suppuration, or ascites.

The *symptoms* in inflammation, as contradistinguished from the signs which mark its presence, require to be traced in reference to 1, Inflammation ; 2, Suppuration ; 3, Gangrene. In acute adhesive inflammation, the most general and uniform symptom is an increase in volume and frequency of the pulse, indicating unhealthy activity of the heart—rigor is frequent; and one important diagnostic symptom, is, "augmented power to bear the loss of blood." Scarcely any of the symptoms characteristic of fever are present. Severe rigor, sometimes often repeated, announces the presence of suppuration, but frequently calls for great skill to distinguish it from intermittent fever. "Sometimes one rigor only occurs; sometimes several rigors occur, at uncertain periods; in other cases the rigors are repeated at such regular quotidian or tertian periods, as to lead to the idea that the case is intermittent fever or ague.

*Gangrene* is depicted in the collapsed condition of the countenance, and of the general system: paleness, and cold, clammy perspiration, and sunken features; a similar condition of the general surface, and of the extremities; sub-delirium; tremor; oppression; sickness; a dry, brown tongue; sordes on the teeth; a feeble, thread-like pulse.

The secretions are more or less altered—the skin becomes drier and hotter—the insensible perspiration being suppressed, and in its place partial and unnatural sweatings occur. The urine is diminished in quantity, and altered in quality, becoming dark, high-coloured, cloudy and turbid, and yields a copious precipitate on the addition of corrosive sublimate.

Other general phenomena are also observable, and consist in the suspension, interruption, or injury of some healthy action or function. The disturbance of circulation may be attended with changes in sensation and motion, and temporary derangement of the intellectual functions. Disturbed respiration, or morbid forms of inspiration and expiration, may also accompany it; as likewise may various derangements of the alimentary functions.

The remarks of Dr. Craigie upon the importance of attending to the functional changes, which take place in inflammation, are so valuable that we need not apologize to our readers for transcribing them entire:—

"It will be found that it is impossible to describe the symptomatic fever of inflammation in general terms, or to limit it to invariable characters; and experience will show, that in one case the functions of circulation and respiration will be most affected; in another the functions of circulation and alimentation will be most affected; and, in a third, the prominent features of the fever will be found in the derangement of sensation and intellect, with injury to the alimentary function. It will also be found that the degree in which one function is affected will often remarkably modify the morbid changes incident to the other. In inflammation of the chest, or its organs, in which the circulation and respiration, and the dependent processes of secretion, &c. are invariably much deranged, the intellect is often perfectly clear, sensation quite correct, and muscular motion regular and healthy; and even the alimentary function is rarely much impaired during the symptomatic fever of pulmonary inflammation.

"On the other hand, when inflammation is seated in the brain or membranes, during which the patient may be delirious, or completely insensible, or affected with various spasmodic motions of the voluntary muscles, or lose the power of moving his limbs entirely,—the motion of the heart may not be quicker than natural, and in some instances is actually slower, and respiration may be in a correspondent state. On other occasions these functions are very considerably affected by the process which is going on in the head; and the patient may die with very laborious breathing and a small fluttering pulse.

"Another instance of this modification of one order of processes in the living body by the condition of another, is presented in the inflammations of the alimentary canal. The morbid process in general produces a direct effect on the functions of this tube; and furred tongue, extreme thirst, squeamishness, loathing and actual vomiting, are the first results of its influence. While this disturbance, however, is occasioned in the alimentary function, that of circulation is often little or not at all affected. The pulse is sometimes not more rapid or harder than natural, and the respiration is quite healthy. There is reason to believe that in such instances, either the distressing sickness, which is known to influence the motion of the heart, prevents that frequent action which is sure to take place in ordinary circumstances,—or the inflammatory act may exist to a certain extent in sundry parts of the alimentary canal, without being followed by much disturbance in the function of circulation."—*Craigie*, p. 393.

III. The Changes in the Condition of Inflammation, according to Dr. Hall, are eight. Resolution—œdema—adhesion—cicatrix—softening—induration—ulceration—suppuration and gangrene. Dr. Craigie enumerates them somewhat differently; according to him, they are—resolution, deranged secretion, effusion, adhesion, hæmorrhagic effusion, softening, induration, hypertrophy, suppuration, ulceration, granulation, cicatrization, and mortification.

1. *Resolution* is the mere subsidence of inflammatory action, and the only proper termination of the process. It is effected by the absorbent power of the minute veins, and the contractile power of the capillaries.

2. The interstitial effusion of limpid albumen or serum, viewed distinctly from the repletion and enlarged size of the capillary and minute vessels, constitutes *œdema*, the *white-swelling* of inflamed parts, sometimes accompanying, sometimes following, the actual inflammation. It frequently remains in the form of a pale and colourless swelling, after the vascular repletion and the consequent redness have disappeared. In one case—inflammation of the larynx—it is frequently the cause of death, obstructing the upper orifice of the trachea and suspending respiration.

3. *Adhesion* is effected by an intervening deposit of coagulable lymph, or albumino-fibrine. When this takes place, uninterrupted by other processes, it is what surgeons designate *union by the first intention*. When other processes intervene, the effect is slower and modified, and cicatrization takes place. Both these occur in all the tissues of the body alike, whether internal or external, whether canals or cavities.

4. In *softening*, or *ramollissement*, the opposite of adhesion takes place—the natural cohesion of the inflamed part is destroyed; it, likewise, occurs in all the tissues; most, in the parenchymatous substances and mucous membranes; least, in serous membranes. In certain tissues, as that of the lung, it is analogous to mortification of other tissues. In others, the brain, for instance, it corresponds to suppuration.

5. *Induration* belongs to chronic, *softening* to acute inflammation, and depends upon the interstitial deposit, probably of coagulable lymph, or albumino-fibrine.

6. Interstitial absorption, whereby the superficies of a part is removed, produces *ulceration*, which is *simple*, or healthy; *spreading*, or phagedænic; and *destructive*, or sloughing. In the external cutaneous, internal mucous, and synovial membranes, it is common; in the serous membranes, rare;—and it obtains the name of *suppuration* when it takes place in parenchymatous substances; and *caries*, in bony textures. It may proceed, or it may yield to an opposite process, as cicatrization. And, as an ulcerating is always an absorbing surface, it may give rise to enlargement of neighbouring glands, and to inflammation of the absorbent vessels. "The affection of the inguinal glands in chancre, and also in gonorrhœa, is a fact familiar to us all. Is there ulceration in all cases of the latter malady thus complicated with bubo?"—*Hall*, p. 11.

7. The observations of Brugmann, Hunter, &c. prove that purulent fluid may be formed without any breach of surface. This is *suppuration*, and one of the most frequent results of inflammation—exhibiting itself in the four varieties of *abscess*, where the pus is enclosed in an orbicular cavity; *fistula*, where it burrows between the adjacent textures; and *infiltration* and *diffusion*, when it gets into the meshes of the cellular membrane, or is spread over the surface upon which it is formed.

8. Inflammation sometimes leads to *gangrene*, and to *sphacelus*; terms, conventionally employed to designate—the former, the condition of the part when on the point of losing its vitality—the latter, that of a part absolutely dead, and ready to pass into a state of decomposition.

"Nothing can illustrate the varied phenomena of inflammation, on a minute scale, better than the variolous pustule; at first, we have simple inflammation—inflammation of a sebaceous gland—redness and tumor; on the third and fourth days we have the effusion of *serum*, a vesicle, the duct of the gland tying down its centre; on the fifth day, we observe the effusion of *pus* around this central point and within the external margin of the vesicle, the intervening space being occupied by transparent serum, and appearing of a red, flesh-colour, well contrasted with the opaque pus, and there is a surrounding *areola* of deep inflammation; on the seventh or eighth day, the serum is entirely replaced by pus; and on the eighth or ninth, the central duct has been absorbed or has sloughed, and the pustule is orbicular. There is also the early effusion of *lymph*; and, at a subsequent period, it is found that a portion of the cutis vera has sloughed. The whole of this series of the phenomena of inflammation is followed by cicatrization, again implying the effusion of lymph."—*Hall*, p. 15.

IV. In treating of *the Nature of Inflammation*, Dr. Hall institutes an inquiry into the condition of the true capillary, the secretory, absorbent, and *newly*-formed vessels, the minute arteries and veins, the larger vessels, the heart, and the blood. From which he concludes that each cause of inflammation induces such a physical effect upon the internal surface of the capillaries as leads to the adherence of the globules of blood to it, and to their ultimate secretion. This stagnation augments as the inflammation increases and becomes more diffused; and seems to constitute the essential character of the disease. Any augmented or diminished action on the application of stimuli, he has never been able to detect.

Obstructed capillary circulation leads to enlargement of the minute arteries—arteries being muscular organs, and muscular organs always augmenting in the ratio of the opposition to be overcome by them in the performance of their functions. The condition of the minute veins is not ascertained.

The secerning vessels are variously affected by the various degrees of inflammatory action—an effusion of serum, marking the lower; a secretion of albumino-fibrine, the higher degrees; and pus, the highest of all.

The functions of the absorbent vessels are not less modified than those of the secerning vessels.

After lymph has been long poured out, it becomes organized, and numerous vessels carrying blood, are observed in it, pursuing a various course: these have been delineated by Monro, Hunter, and Lobstein, as seen in cicatrix, in portions of pendulous coagulable lymph, in layers of lymph, and in coagula of blood.

The enlargement of the blood-vessels is not confined to the minute arteries—the larger arteries, in the immediate vicinity of the inflamed part, also enlarge. It is not certainly known how far this enlarged condition of the arteries extends from the seat of inflammation, but that the inflammation of a part affects, not only the arteries and veins in its vicinity, but also the heart, is gathered from facts like these:—The pulse of the radial artery, leading to an inflamed hand, is more forcible than that of the other wrist; the veins leading from the inflamed hand, yield their blood more freely than the similar veins of the other arm; the heart also beats with an augmented impulse and greater frequency.

"The blood is well known to undergo considerable changes in inflammation: the appearances of cupping and of buff, of the blood drawn from a vein, are sufficient evidence of this fact; if the same appearances have not been observed so familiarly upon arterial blood, it is probably because arteriotomy is much less frequently performed than venesection."—*Hall*, p. 21.

In Dr. Hall's chapter on this very interesting part of the subject before us, he has equally aimed to preserve facts and to discard conjectures, rightly observing, that—The condition of medical science still requires this separation of what is ascertained from what is only imagined—of the true from the false: to discover the former, and to detect the latter, are equally benefits conferred upon our profession. A sentence, which it would be well for every medical student to carry with him throughout his curriculum—and one, which every medical author would do well to set before his eyes whenever he takes the pen into his hand. Medicine, perhaps, more than any other science, if we except at the present time political economy and divinity, is inundated with conjectures and vain imaginations, and crude and undigested fancies, the abortions of prolific but feeble minds—and facts, comparatively few, come to be disregarded because of the superfluities of fiction, than which, there is nothing more evil, more dangerous, or more destructive of true science.

V. The Modifications of Inflammation, are most important and interesting, as these arise out of the varieties of texture, or are produced by differences in the conditions of the system.

*Inflammation of the serous membranes* is marked by redness, and found to consist in points, stars, and arborescent forms, arising, 1st, from enlarged vessels, and, 2d, from extravasated portions of blood. Dryness, from checked secretion, is rare; augmented effusion from the surface of the membrane, more general. This effusion consists of serum, coagulable lymph, albumino-fibrine in *layers*, or adhesions, pus or puriform fluid; sanguineous serum. An important, although negative character of inflammation of the serous membrane is, that it seldom leads to ulceration.

In inflammation of the mucous membrane there is redness, injection, enlarged bloodvessels, increased secretion of mucus, at first transparent, afterwards opaque and puriform, re-assuming its transparence as the inflammation subsides. The exudation of coagulable lymph is rarely seen, although we have instances of it in the trachea in croup; and in the uterus, in dysmenorrhœa, when it forms, in each case, a false membrane.

Corresponding as inflammation of the mucous membranes does in these respects to that of the serous membranes, it is, in others, as diametrically opposed. Inflamed mucous membranes *soften* and *ulcerate*—events happily uncommon in inflamed serous membranes.

" Suppose an abscess in the liver. It enlarges; it proceeds to evacuate itself; this may be effected externally, in the hypochondrium; internally, into the intestines, or through the lungs into the bronchia. In the first case, adhesive inflammation unites the two contiguous portions of peritonæum, and the subsequent ulcerative process pierces through these two folds of membrane with the intervening layer of albumino-fibrine—and then through the external integuments. The cavity of the abdomen is protected and preserved from an effusion of pus, which would immediately induce a terrible and fatal peritonitis! In the second case, similar phenomena occur, and the abscess finds an issue into the intestine, the abdomen being still protected and preserved as before. In the third case, the two contiguous peritoneal surfaces first, and then the two adjacent pleural surfaces, unite by albumino-fibrous adhesions; and, lastly, the ulcerative process proceeds to open a way for the pus through these adherent membranes, the intervening diaphragm, the cellular tissue, and the bronchial parietes, and the pus is eventually expectorated, the cavity of the abdomen and that of the pleura being equally preserved!"—*Hall*, p. 28-9.

Mr. Travers tied the duodenum of a dog, so as completely to obstruct the passage. On the two following days the animal was sick, and his respiration hurried. On the 5th day he passed a copious stool of the same appearance as the fluid discharged by vomiting. From this time the sickness ceased, the breathing became natural, he fed and digested his food—the cure was established by the fifteenth day, and he was then killed. On examination, the lacteal system was well displayed. The folds of a portion of omentum contiguous to the strictured intestine adhered to it. A slight depression was observed in the circumference of the gut—which was then carefully laid open—the villi were turgid with chyle—the villous surface more vascular and deeper-coloured than usual. A transverse fissure marked the seat of the ligature. The edges of the section were distinctly everted, and the appearance corresponded with that of the union by suture.

In another dog, a fold of ileum was strangulated, a little above the angle. The strangulated piece below it was then cut off, and the cut extremities joined by ligature. The wound was sewed up, the animal not appearing to suffer materially—the second and third days he was sick and vomited bile, but drank a little milk and water. On the 4th day, passed a solid stool.

In a month, was perfectly well, and shot. The external wound was healed. There was no appearance of disease in the abdomen—and but few adhesions of the peritoneum. The ileon lay thus  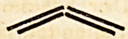 upon the vertebræ. At the internal angles the sides adhered to each other. The opposite was closed by adhesions to the omentum and neighbouring intestine. Upon laying open the tube, it appeared that the ligature and the ends of the gut had been discharged through the canal. At one point, the line of union was scarcely completed; and there appeared a little cyst, like an abscess, communicating with the tube, in which the tied ends of the gut had been lodged previously to their being voided.

"In all these cases," and in the similar ones of hernia, and of intussusception, "the contiguous points of serous membrane unite by the effusion of albumino-fibrine; the interior tissues, with the mucous membrane, are severed by the ulcerative process. The cavity of the peritoneum is *guarded* from the irruption of the fæcal matters by *adhesive* inflammation; whilst the canal of the intestine is *preserved entire* by the *ulcerative!* Now let us suppose these properties of the serous and mucous membranes reversed! Every inflammation of the former would tend to ulceration and abscess; every inflammation of the latter, to close a canal!"—*Hall*, p. 31.

Inflammation is also modified by the different conditions of the system. In rubeola, for instance, the phlogistic diathesis prevails. In scarlatina, the character of the local inflammation and its attendant fever, is frequently typhoid. And changes take place corresponding to these differences. The changes which take place in inflammation occurring in the phlogistic diathesis, as has already been shown from Dr. Hall's work, are aptly illustrated by the varioloid inflammation, in the *distinct* form; that which takes place when the typhoid type is present, receives a similar illustration from the pen of our author in the *confluent* variety—the papulæ are less hard and elevated; the serum and pus are less distinctly characterized, and resemble an undefined, and sometimes bloody, *sanies;* the progress, the circular and orbicular forms, the periods, the termination of the eruption, are less marked, less distinct; and there is a greater disposition to slough, and consequently, to scars.

We have been led into so full an analysis of this subject, as treated by Drs. Hall and Craigie, that we have little space left for considering the chapters devoted by the former to "the distinctions between inflammation and irritation and congestion"—"the influence of inflammation"—and "the diffusion of inflammation"—and must very briefly notice that which treats *of inflammation as a curative means.* Without it, the art of surgery could not exist. Every operation implies the resourses of Nature in healing divided parts. As a curative measure it is employed by Nature and Art. By the former, in apoplexy, in the formation of an artificial anus, and in conducting pus from an hepatic abscess to the surface;—by the latter, in the cure of artificial anus as devised by Dupuytren, in the cure of hydrocele by injection—in that of prolapsus uteri—and in the treatment of nævus, &c. —the two latter applications of it are claimed by Dr. H. as inventions of his own.

VI. The Treatment of Inflammation receives no additions of any consequence from either of the writers before us—to the remedies more especially called for in internal inflammation, viz. blood-letting, mercury, and tartrate of antimony, Dr. Hall devotes particular attention. We pass over the intermediate chapters on tubercle, melanosis, fever, &c. to notice these.

*On Blood-letting.* It is a remarkable fact, that "if several patients of similar strength and constitution, but affected by dissimilar diseases, be respectively placed in the erect position and bled to deliquium, they will be found to lose very various quantities of blood," one will bear the loss of 50, 60, or even 70 ounces, without syncope—another will not endure to lose four ounces.

The rationale appears to be, that, "different diseases induce in the constitution different powers or susceptibilities in regard to the effects of loss of blood. Each disease seems to have its own virtue in this respect; this is determined by placing the patient perfectly erect, and bleeding to incipient syncope: the quantity of blood which flows is the measure of the protective influence of the disease in one class of cases, and of its influence in superintending a susceptibility to the effects of loss of blood on the other. In cases in which it is doubtful whether the pain or other local affection be the effect of inflammation or of irritation, the question is immediately determined *by placing the patient upright and looking upwards, and bleeding to incipient syncope:* in inflammation much blood flows; in irritation very little. The violence of the disease, the powers of the system, and the due measure of the remedy, are determined at the same time. *There is, in my opinion, no single fact in physic of equal importance and value, in the diagnosis of acute diseases, and in the use of a powerful remedy.*"—*Hall,* 79-80.

An interesting scale of diseases may be formed representing these properties.

Persons in health, of moderate strength, will generally faint if bled in the erect posture, on taking fifteen ounces of blood. Dr. H. has known 70 ozs. to be taken in the sitting posture, in the tendency to apoplexy without syncope! but the case is an extreme one. Patients with meningitis, encephalitis, pleuritis, or pneumonia, frequently lose 35 ounces of blood without fainting. In *bronchitis,* little more is borne to be lost than in health. A stout person in *fever* will frequently faint on losing 10, 12, or 14 ounces of blood. In *intestinal irritation,* with urgent symptoms, even the abstraction of nine or ten ounces will generally induce deliquium. In *delirium tremens* or *puerperal delirium,* the patient soon faints. And the same thing is observed in cases of violent reaction, arising from loss of blood. In dyspepsia, hysteria, and chlorosis, the susceptibility to syncope is very great, and Dr. H. has known a patient of good strength, affected with cholera, faint on taking four ounces of blood, who had previously lost, under the influence of inflamed mamma, 20 ounces without faintness. *Paralysis* from *laceration of the brain,* and *apoplexy* from concussion, before reaction takes place, or inflammation is established, are cases also of diminished tolerance. It must also be carefully noticed that cases of accident do not bear the loss of blood like those of inflammation.

These facts afford:—1. A rule for blood-letting, in all cases in which it requires to be fully instituted. 2. A guard at once against inefficient and undue blood-letting. And 3. A source of diagnosis.

The *rule* is suited to the degree as well as the duration of the disease; and is not less adapted to those most frequent of all events, *mixed* cases—inflammation and irritation conjoined.

"It is difficult to say whether more injury has been done by an undue or by an inefficient use of the lancet. In inflammation we must bleed fully—in irritation we must bleed cautiously. Inefficient blood-letting in the former, and undue blood-letting in the latter, are alike dangerous or even fatal to the patient; from both extremes we are guarded by the rule which I propose. By directing the patient to be placed in the erect position, and bled to incipient deliquium, we shall often take much more blood than we should have ventured to *prescribe* to be taken in inflammation, and very much less than we might be supposed to direct in irritation; and in both these cases the rule conducts to the only safe mode of treatment. And, if much blood has flowed before the occurrence of syncope, inflammation must be suspected; if little, we must suspect that, however similar the symptoms, the case is in fact of a different nature—perhaps irritation—perhaps exhaustion."—*Hall*, p. 81.

He has also found that, in every case in which early syncope occurs from blood-letting, the more remote effects of loss of blood, as reaction, or sinking, are also very liable to occur; and it is in these cases that sudden dissolution has always followed the use of the lancet. There is, in every point of view, intolerance of loss of blood. The reverse of all this obtains in inflammation, which seems to be incompatible, to a certain degree, with the effects of loss of blood; these are, on the other hand, very apt to supervene as the inflammatory action subsides.

Dr. Hall very wisely solicits the co-operation of the profession in the further investigation of this subject, not imagining his "*rules*" to be without exceptions, and rightly deeming it "as important that these should be pointed out, as that the rule itself should be established." He makes mention, himself, of two such. In some cases of fever requiring blood-letting, the patient cannot support the erect position: in such a case the arm must be first prepared; the vein should then be promptly opened; and then the patient should be gently raised and *supported* in the upright position, carefully avoiding all muscular effort. On the other hand, in the case of congestion of the brain from exhaustion, there is not such early syncope from blood-letting as might be expected; and yet it is obvious that the system cannot bear the loss of blood.

"Two objections have been made in reference to this rule for the administration of blood-letting: the *first* is, that in some cases not inflammatory, more blood might be taken than the patient could bear to lose in order to institute the test; my reply is, that such cases are not *included* in my proposition, which only relates to cases in which blood-letting *is required to be* FULLY instituted." The 2d is, that in some cases *more* blood ought to be taken than would flow before syncope is induced. I greatly doubt this assertion; I think it replete with *peril;* but if it be true, let the patient be replaced in the recumbent posture, wait a few minutes, and again let the blood flow; we have at least ascertained the state of tolerance of loss of blood, and this fact will *guide* us in determining *how much more* blood may be withdrawn; it is a fact *added;* it is *knowledge* substituted for what *must* otherwise be ignorance." P. 84.

The results of Dr. Hall's investigations appear in a table, which we shall add to the pages of this review, fully agreeing with its very able author, that—No one *can* pass the eye over it without being impressed with the value and importance of the facts it displays, with the diagnostic, the guide, the guard, which it affords.

| | |
|---|---|
| I. AUGMENTED TOLERANCE. Represented by the mean quantity of blood which flows before incipient syncope. | |
| I. Congestion of the brain. | |
| 1. Tendency to apoplexy | ℥XL.—L. |
| 2. Apoplexy from congestion | |
| II. Inflammation of the serous membranes. | |
| 1. Arachnitis | ℥XXX.—LX. |
| 2. Pleuritis | |
| 3. Peritonitis | |
| 4. Inflammation of the synovial membrane, and of the fibrous textures of joints | |
| III. Inflammation of the parenchyma of organs. | |
| 1. Of the substance of the brain | ℥XXX. |
| 2. Pneumonia | |
| 3. Hepatitis | |
| 4. Inflammation of the mamma, &c. | |
| IV. Inflammation of the skin and mucous membranes. | |
| 1. Erysipelas | ℥XVI. |
| 2. Bronchitis | |
| 3. Dysenteria | |
| II. HEALTHY TOLERANCE. This depends on the age, sex, strength, &c., and on the degree of thickness of the parietes of the heart, and is about | ℥XV. |
| III. DIMINISHED TOLERANCE. | |
| 1. Fevers and eruptive fevers | ℥XII.—XIV. |
| 2. Delirium tremens and puerperal delirium | ℥X.—XII. |
| 3. Laceration, or concussion of the brain | ℥VIII.—X. |
| 4. Accidents before the establishment of inflammation | |
| 5. Intestinal irritation | |
| 6. Dyspepsia. Chlorosis | ℥VIII. |
| 7. Cholera | ℥VI. |

Dr. Hall quotes largely from Mr. Wardrop's lectures in confirmation of the views published by him, but with an expression of sorrow, which is meant to convey a gentle hint to the latter gentleman, that he has set forth as his own what he ought to have acknowledged as coming from our author. Our readers will recollect that in the Number of the Med.-Chir. Review, for April, we had occasion to record a similar complaint preferred by Mr. Mayo against Dr. Hall. It is the penalty which an original thinker must be content to pay for the exclusive merit of originality, that his thoughts will be appropriated by those who were never capable of conceiving them—and his improvements will be more widely adopted than generally acknowledged. The great reward of all such, and that to which we would fain entreat them to turn from ungenerous rivalry, and piratical plagiarism, is the high and noble consciousness of having " done the state some service," whether known and acknowledged or not. Men may be impeded in a career of extensive and extending usefulness, by condescending to defend their own claims to the merit of discoveries, or inventions—when silence would be the severest punishment they could inflict on those who " filch from them their good name;" and leave themselves free to prosecute their course, right onward to the sure guerdon of patient merit—which is certain to be dispensed by that almost only dealer in even-handed justice—Time.

With these remarks we would conclude our notice of Dr. Hall's chapters on Inflammation and Blood-leting, but must add a couple of most interesting cases. A gentleman, a few miles out of London, was seized with an affection of the head. Dr. Hall was sent for, and found that he had been bled early, and had fainted speedily from loss of blood. " The case was long one of great anxiety. Another physician was consulted. It was supposed to be ' ramollissement.' Confiding in the diagnosis afforded by the *early* syncope, in the *early* stage of the disease, from blood-letting, I continued to hold out hope of recovery. The patient did recover! Now, the hope felt and held out, in this case, flowed almost entirely from the confidence reposed in the diagnostic afforded by the event of the blood-letting!" Dr. Hall was further consulted by a medical man, who feared peritonitis, in the case of his wife. There had been previous attacks of the same kind; there were great pain and tenderness of the abdomen; the tongue was loaded: blood-letting in the upright position, was prescribed; the patient, though young and stout, fainted on losing 8ozs. From this moment the diagnosis was clear—the treatment unequivocally indicated—the recovery uninterrupted.

With the final observation of our author that, in all cases where there is great tolerance of blood, blood-letting is remedial and safe—in all cases in which there is intolerance, it is proportionably of doubtful efficacy, and replete with danger; we conclude, for the present, out review of these volumes. As they are both written for the student and young medical practitioner, we are happy to have it in our power to recommend both to those for whom they were designed. Dr. Hall's is, like most that he writes, excellent; so much so, that its brevity is the chief fault we have to find with it. Dr. Craigie's is a very valuable compendium of the present knowledge possessed by the profession, but it is heavy in the perusal from being as much too long as the other is too short. If Dr. Hall would fill up his own outlines fully, he would render an inestimable service to the profession and the public. And, on the other hand, if Dr. Craigie would employ some of his pupils at the not unprofitable drudgery of stripping his important facts of the unnecessary verbiage in which he has introduced them to his readers, he would reap the benefit of it, by his volumes being much more extensively circulated, and thereby rendered much more generally useful.

No pupil in either of their classes, should be without these works—as a text book, interleaved, and to be filled up by the diligence of the student, from the oral lectures of the author, Dr. Hall's will be indispensable to his own pupils—if they would profit by the teaching of such a master, who, if he lecture, as well as he writes, will command attention, and say little that is not worthy of being committed to memory.

There still remains a great want in the study of medicine, which these volumes no more supply than others that have gone before—and which, is only partially supplied by our professional cyclopædists. We mean a regular course of instruction, which shall preserve our students from desultory studies, and from partiality in the choice of subjects for study. Rome, however, was not built in a day, and when we compare the present state of the medical classes in London, with those of our young days, we take courage from the prospect they open, to believe that the march of improvement in medical education is rapidly progressing to that point, whereat, all who are studious may learn—all who are diligent may acquire knowledge—and all who are solicitous to improve in their profession will find facilities afforded them to do so, and the hinderances to their doing so, taken out of the way. There is, indeed, no royal road to knowledge—but there are many impediments in the beaten paths, and many of those paths are unnecessarily rugged, as well as circuitous. Never mind! We shall have some Mac Adam to repair the roads for us in medicine by and bye—and then, we see no just cause or impediment why, when the railways are once laid down, we should not travel with the rapidity of a steam-carriage, along the beaten track, from one subject to another, through every department of science.

